# Dietary Protein Levels in Isoenergetic Diets Affect the Performance, Nutrient Utilization and Retention of Nitrogen and Amino Acids of *Hermetia illucens* (L.) (Diptera: Stratiomyidae) Larvae

**DOI:** 10.3390/insects16030240

**Published:** 2025-02-25

**Authors:** Laura Schneider, Benson Kisinga, Nathalie Stoehr, Stefan Cord-Landwehr, Elmar Schulte-Geldermann, Bruno M. Moerschbacher, Klaus Eder, Rajesh Jha, Georg Dusel

**Affiliations:** 1 Department of Life Sciences and Engineering, University of Applied Sciences Bingen, 55411 Bingen am Rhein, Germany; 2 Institute of Animal Nutrition and Nutrition Physiology, Justus-Liebig-University Giessen, 35392 Giessen, Germany; 3 Institute for Biology and Biotechnology of Plants, University of Münster, 48143 Münster, Germany; 4 Center for Sustainable Food Systems, Justus-Liebig-University Giessen, 35390 Giessen, Germany; 5 Department of Human Nutrition, Food and Animal Sciences, College of Tropical Agriculture and Human Resources, University of Hawaii at Manoa, Honolulu, HI 96822, USA

**Keywords:** black soldier fly larvae, insect nutrition, amino acids, retention

## Abstract

Black soldier fly, *H. illucens* larvae, efficiently convert low-value organic substrates into high-value products, offering solutions to global challenges in sustainable food production and biotechnology. This study investigated the impact of dietary protein levels (10%, 14%, 16%, and 20% crude protein, CP) on BSFL growth, nutrient utilization, and energy retention using isoenergetic diets (18.5 ± 0.3 MJ/kg dry matter) under commercial-scale conditions. Larvae were harvested after 8 days of feeding, with 5 replicates per treatment. Optimal growth performance and feed conversion ratios were observed in larvae fed 14% CP diet, with a quadratic relationship between dietary CP and biomass gain (*p* < 0.001, R^2^ = 0.870). Ash and calcium deposition peaked in CP20-fed larvae and were lowest in CP14-fed larvae. Phosphorus and glucosamine deposition remained unaffected, while chitin deposition correlated positively with larval weight. Nitrogen and amino acid retention were highest in CP14-fed larvae but reduced in CP20-fed larvae (*p* < 0.001, R^2^ = 0.573–0.902). CP10-fed larvae showed impaired growth and nitrogen deposition but increased fat deposition. These findings establish the CP14 diet as the optimal formulation for scalable BSFL production, providing critical insights into dietary protein effects on BSFL physiology and enabling the development of efficient feeding strategies for industrial-scale farming.

## Simple Summary

Black soldier fly, *Hermetia illucens* (L.), larvae (BSFL) efficiently convert organic low-value substrates into high-value proteins, lipids, and chitin, offering solutions to global challenges in sustainable food production and biotechnological innovation. While BSFL-derived products, such as BSFL protein meal for animal feed, have been widely studied, the nutritional requirements of BSFL themselves remain insufficiently explored, and knowledge in this area is still limited. This study investigates the impact of dietary protein levels (10%, 14%, 16%, and 20% crude protein, CP) in isoenergetic diets on BSFL growth performance and nutrient utilization. Our findings indicate that larvae fed diets containing 14% protein exhibited the highest growth rates and the most efficient conversion of nutrients. In contrast, larvae on a high-protein diet (20%) accumulated the highest levels of minerals, such as calcium, yet exhibited a reduced retention of nitrogen and amino acids. On the other hand, low-protein diets (10%) led to decreased larval growth, lower chitin and higher fat deposition in the larvae. These findings highlight the importance of balancing dietary protein to optimize BSFL farming and improve sustainable production strategies.

## 1. Introduction

As the global population grows and urbanization, climate change, and soil degradation intensify, essential resources like water, energy, and arable land are under increasing pressure. These challenges, along with rising life expectancy and climate uncertainties, make it increasingly difficult to sustainably produce enough food to feed the world [1,2]. To meet this escalating demand, society must look beyond conventional sources and embrace innovative, alternative resource streams that are both efficient and sustainable.

Black soldier fly *H. illucens* larvae represent a promising solution, capable of converting low-value organic substrates—including food waste, livestock manure, and food production side streams—into high-value products such as proteins, lipids, and chitin. These bioconversion capabilities position BSFL as a sustainable resource for animal feed and biotechnological applications [3,4,5,6,7,8,9,10]. However, while interest in BSFL farming is growing, critical knowledge gaps remain regarding their nutritional requirements, particularly optimal dietary protein levels, which are crucial for achieving sustainable and profitable production [11,12,13].

Dietary protein is essential for larval growth, biomass development, digestion, immune function, and hormone regulation [14,15]. Protein supplies essential amino acids (EAA) required for growth. When protein sources are deficient in EAA, larval development can be hampered [15,16]. Conversely, excessive dietary protein levels increase feed costs and contribute to environmental pollution through nitrogen excretion. Optimizing protein intake not only improves feed conversion efficiency but also reduces the environmental footprint of BSFL farming [16,17,18,19]. Existing studies highlight the importance of a balanced protein-to-energy ratio in larval diets, but the role of EAA, which are critical for growth and cannot be synthesized by larvae, remains underexplored [20,21,22,23].

For instance, Tomberlin et al. [21] identified 10 essential amino acids for BSFL, including arginine, histidine, isoleucine, leucine, lysine, methionine, phenylalanine, threonine, tryptophan, and valine. These amino acids play key roles in protein synthesis and physiological functions, such as arginine’s involvement in digestion via nitric oxide production. A lack of EAA can significantly reduce larval biomass [23]. Additionally, the nutritional quality of BSFL products is influenced by larval feeding substrates, which affect growth rates, body composition, and survival [19,24]. For example, high-carbohydrate but low-protein diets may result in suboptimal larval growth due to EAA deficiencies.

This study aims to provide insights into optimizing BSFL nutrition by identifying the optimal protein concentration in isoenergetic diets. These findings will enhance larval growth performance and nutrient utilization, improve resource efficiency, and promote environmentally sustainable insect farming practices.

## 2. Materials and Methods

### 2.1. Experimental Diets

Based on the experiments conducted in our laboratory and according to previous studies [13] the study tested five different experimental diets on BSFL: four isoenergetic diets with increasing crude protein levels (10%, CP10; 14%, CP14; 16%, CP16; 20%, CP20). The standard Gainesville diet [25] was used as a control for environmental conditions (Table 1). Prior to the preparation of the experimental diets, the dry matter content of all raw materials was quantified using a precision moisture analyzer (Radwag MA 200/1.X2.IC.A, Radon, Poland). The substrates were weighed and mixed with warm tap water (27 °C) to achieve a 75% moisture content. After preparation, a 150 g representative sample was collected from each container and analyzed for nutrient concentrations (Table 1 and Table 2).

### 2.2. Rearing Conditions

The BSF colony was obtained from a commercial breeding farm (madebymade GmbH, Pegau, Germany). Newly hatched larvae were reared on chicken feed until they were 6 days old larvae (DOL). Rearing continued in the laboratory of the Department of Animal Nutrition (University of Applied Sciences, Bingen), where larvae were sieved through 2.4 mm and 1.0 mm sieves to standardize size, removing oversized and undersized larvae. Three 1 g samples were taken and counted manually to determine the average weight of a starter larvae (7.9 ± 0.2 mg) and calculate the total mass of larvae for each container.

For the experiment, 2765 g of 6DOL were placed in a controlled climate chamber (27 ± 1.5 °C, 55 ± 5% RH) and randomly assigned to substrates. Larvae were grouped into batches of 14,000 individuals and housed in containers (40 cm × 60 cm × 12 cm; 2.1 cm^3^/larvae), each containing 2800 g dry mass of substrate (0.2 g DM/larvae; 0.8 g FM/larvae) and 110 g of 6DOL. The larvae were kept in containers for 8 days, during which no additional feeding or mixing was carried out. In all examined container units, fewer than 5% of the larvae reached the prepupae stage, suggesting that the larvae were nearly at the same developmental stage during the observation period. The containers were arranged in a vertically stacked system, following standard rearing practices, and were rotated once daily at the same time within the climate chamber to ensure homogeneous environmental conditions.

### 2.3. Sample Collection

At harvest, larvae were separated by sieving. Test conditions were based on previous experiments to ensure proper feed consumption and dry matter collection. Larvae were manually separated, rinsed, gently dried, and weighed. Fresh matter (FM) was measured by weighing the samples immediately after collection. Substrates and larvae were stored at −18 °C. Before lyophilization, the moisture content was measured using a thermogravimetric method (M35 Moisture Analyzer, Sartorius, Göttingen, Germany). All samples were freeze-dried within 36 h using a 20 L freeze-dryer (CHRIST, Osterode am Harz, Germany) and subsequently ground to pass through a 1 mm sieve (ZM 100, RETSCH, GmbH, Haan, Germany). For the analyses of gross energy, protein, AA, fat, neutral detergent fiber (NDF), ash as well as calcium and phosphorus 1 g of lyophilized substrate and larvae were used.

### 2.4. Analysis of Diet and Larval Composition

Samples were analyzed according to official standardized methods of the Association of German Agricultural Analytic and Research Institutes [26]. Gross energy (GE) was measured via bomb calorimetry (IKA C 5000, Staufen, Germany). Dry matter determination was performed according to method 3.1 [26], in which a 1 g sample was dried at 103 °C for 4 h, with timing starting once the temperature reached 103 °C. After cooling in a desiccator, the sample was weighed. Nitrogen concentrations of substrates and larvae were determined using a nitrogen determinator (Leco Corporation, St. Joseph, MI, USA) by the Dumas method [26,27]. AA concentrations were determined by 24-h liquidhydrolysis at 110 °C in 6 mol/L HCl followed by analysis of 16 amino acids using the Waters AccQTag Ultra chemistry on a Waters Acquity UPLC (Waters Corporation, Milford, MA, USA) as described by Wang et al. [27]. 

Crude protein (CP) concentration of substrates was calculated by multiplying the total nitrogen content by 6.25. To account for non-protein nitrogen, the protein content of larvae was further corrected using the formula CPcor = N × 4.76, as described in [17]. Ether extracts were analyzed according to 5.11 [26] and extraction was subsequently performed using petroleum ether in a Behr E6 system (Behr Labor-Technik, Düsseldorf, Germany). Ash represents the inorganic mineral content of the sample. It was determined by pre-washing the sample with a Bunsen burner and incinerating it twice at 550 °C for 6 h in a muffle furnace, per method 8.1 [26]. The residue remaining after combustion was measured to determine the crude ash content. NDF was determined by methods 6.5.1 and 6.5.2 [26], using α-amylase for NDF (Fibertherm FT 12, Gerhardt, Germany). NDF were expressed without residual ash. Calcium and phosphorus were measured by ICP-OES (Quantima, GBC Scientific Equipment, Melbourne, Vic, Australia; according to method 10.8.2) [26]. Starch was analyzed by colourimetry (Roche CobasMira S, Basel, Switzerland; Randox GLUC-PAP Kit, Crumlin, UK) after enzymatic hydrolysis (Merck, 10115-5G-F, Darmstadt, Germany) according to method 7.2.1 [26].

### 2.5. Determination of Chitin and Glucosamine

The chitin/chitosan content in the insect samples was quantified using the method by Urs et al. [28] with a few modifications. To account for the complexity of the insect samples, 5 mg freeze-dried insect samples were used instead of 1 mg. The remaining steps were carried out as previously described. The samples were suspended in 0.5 mL of 6% KOH and incubated for 90 min at 80 °C and 700 rpm to break down the crystalline structure within the samples. Afterwards, the samples underwent centrifugation at 14,000× *g* for 10 min at 4 °C to remove the KOH. The resulting pellet was washed twice with 1 mL of phosphate-buffered saline (pH 7.4). To further break down the material into smaller particles, the pellet containing the chitin and chitosan was resuspended in 250 µL of double-distilled water and homogenized using bead-beating. To fully convert all glucosamine (GlcN) units in the chitin/chitosan into N-acetylglucosamine (GlcNAc) units, the pH of the sample was first adjusted to a pH of approximately 8.5 by adding 0.5 mL of 1 M NaHCO_3_. Next, 50 μL of the isotopically labelled [^2^H_6_]acetic anhydride (Sigma-Aldrich, St. Louis, MO, USA) was added. The reaction mixture was incubated at 30 °C for 48 h with constant shaking at 500 rpm. After incubating the samples for 48 h at 30 °C with gentle shaking, they were freeze-dried, resuspended in 500 μL of water, and filtered using a centrifugal filter modified PES 3K (VWR International, Radnor, PA, USA). A double-isotopically labelled internal standard R*1 (0.1 g/L) was mixed with the filtrate in a 1:1 ratio to enable the quantification of the GlcNAc units via LC-MS according to Urs et al. [28].

### 2.6. Calculations

To determine individual larval live weight, 100 larvae were randomly selected from each container and weighed daily (±0.001 g; KERN ADB/ADJ 200-4, Balingen, Germany) until the end of the study. The larval survival rate was determined by calculating the ratio of the final larval count to the initially estimated number of BSFL, following the method of Guillaume et al. [29]. Larval biomass gain was measured as the difference between the total larval mass per container at harvest and the initial larval mass. The feed conversion ratio (FCR) was calculated as the ratio of distributed substrate to total larval biomass gain. Additionally, the nitrogen conversion ratio (NCR = supply of nitrogen in g per 100 g larval gain), gross energy conversion ratio (GECR = supply of GE in MJ per 100 g larval gain), and total amino acid conversion ratio (TAACR = supply of total amino acids in g per 100 g larval gain) were evaluated. The conversion parameters were determined in fresh matter (FM) and dry matter (DM). Conversion parameter in FM means distributed feed in 88% DM divided by larval gain in FM. Conversion parameters in DM were calculated as distributed feed in 88% DM divided by larval gain in DM.

To assess the efficiency of larvae in consuming and converting nutrients into biomass, the deposition was calculated as the product of biomass gain (g/d) multiplied with the nutrient concentration (g/kg), divided by the weight of 1000 larvae. Nutrient retention (Equation (1)) per container was calculated as the total mass of nutrients in larvae in DM divided by the total mass of nutrients in the feeding substrates in DM, according to Seyedalmoosavi et al. [30].

Retention [% DM] = Larval gain (g)/Substrate supply (g) × 100(1)

### 2.7. Statistical Analyses

The experimental unit was the rearing container (*n* = 5 replicate rearing containers per diet). Data were analyzed using IBM SPSS Statistics (Version 29) with ANOVA, followed by the Tukey HSD post hoc test for multiple comparisons between dietary treatments. The dataset included larval weight, survival rate, biomass gain, and conversion ratios as well as chemical analyses. All parameters were tested for normality using the Shapiro–Wilk test and for homoscedasticity using Levene’s test. When normal distribution was only achieved after a log transformation, the log-transformed data were used for statistical analysis. Further, a curve fitting linear and nonlinear regression analysis was performed. Statistical significance was set at *p* ≤ 0.05, and data are presented as means with their standard error (SE). The control diet was not included in the statistical analysis.

## 3. Results

### 3.1. Growth Performance and Bioconversion

**Figure 1:**
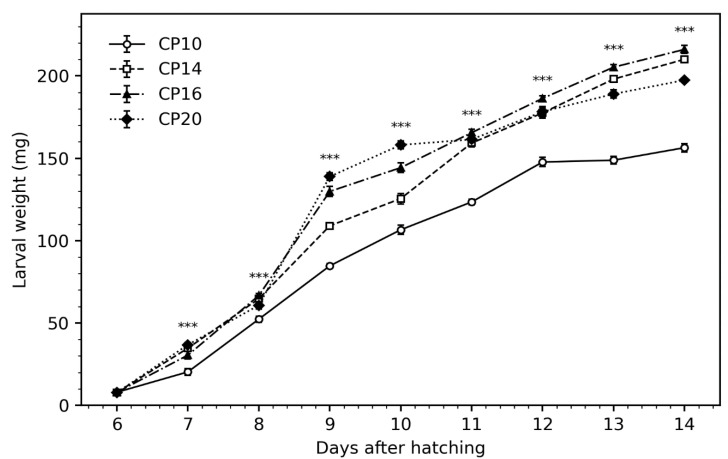
Development of larval weight. Data are presented as means ± standard error (*n* = 5/diet); *** indicates the effect of dietary treatment (*p* < 0.001).

At day 6, the mean larval weight was 7.9 ± 0.2 mg, with no significant differences. Significant differences in larval weight were observed starting from day 7 (Figure 1). Larvae fed with CP20 reached the highest weights between days 7 and 10. In comparison, CP16-fed BSFL showed significantly higher larval weights compared to CP-fed BSFL, although this difference was only evident until day 10. (*p* < 0.001, Figure 1). On day 14, the final larval weights ranged from 157.2 ± 0.2 mg for the CP10 group to 215.7 ± 0.2 mg for the CP16 group (*p* < 0.001, R^2^ = 0.783, Figure 1). The CON-fed larvae weighed 170.2 ± 0.3 mg on day 14. The survival rate of the larvae was not affected by diets (Table 3). The biomass gain of BSFL was influenced by dietary protein levels, with the highest biomass gains (in fresh and dry matter as well) observed in larvae fed the CP16 and CP14, compared to larvae fed the CP10 diet (*p* < 0.001, R^2^ = 0.870 and R^2^ =0.886, Table 3).

Feed conversion ratio (FCR) on a fresh matter basis decreased with higher protein levels, with the lowest values recorded for CP16- and CP14-fed BSFL and the highest for CP10-fed BSFL (*p* < 0.001, R^2^ =0.865, Table 3). A similar trend was observed for FCR on a dry matter basis, where larvae reared on CP14 had the lowest value compared to larvae fed CP10 (*p* < 0.001, R^2^ =0.749, Table 3). Gross energy conversion ratio (GECR) on both fresh and dry matter bases was highest for CP10-fed BSFL compared to BSFL fed with other dietary treatments (*p* < 0.001, R^2^ = 0.859 and R^2^ = 0.747, respectively). Nitrogen conversion ratio (NCR) increased linearly with higher dietary protein concentrations, with the highest value in CP20-fed BSFL and the lowest in CP10-fed BSFL on both fresh and dry matter bases (*p* < 0.001, R^2^ = 0.921 and R^2^ = 0.909, Table 3). The total amino acid conversion (TAACR) showed a marked increase with higher dietary protein levels. On a fresh matter basis, TAACR was 65% higher in larvae fed CP20 compared to CP10. Similarly, on a dry matter basis, TAACR increased by 81% in CP20-fed larvae relative to CP10 (*p* < 0.001; R^2^ = 0.875 and R^2^ = 0.893, Table 3).

### 3.2. Nutrient, Mineral, and Amino Acid Composition

Table 4 presents the analyzed nutrient composition of BSFL dry biomass. Larval water concentration showed no significant differences among treatments. Gross energy concentration similarly exhibited no significant differences. Larval protein concentration was strongly affected by dietary protein levels. The lowest protein concentration was observed in the larvae fed the CP10 diet, compared to all other diets (*p* < 0.001; R^2^ = 0.828, Table 4). Fat concentration in larval dry biomass also displayed a significant quadratic response to dietary protein levels. The highest fat concentration was recorded in larvae fed the CP10 diet, while the lowest was analyzed in the CP14-fed group (*p* < 0.001; R^2^ = 0.693, Table 4). In contrast, ash concentration increased linearly with higher dietary protein levels, with the highest ash concentration found in larvae fed the CP20 diet compared to CP10 group (*p* < 0.001; R^2^ = 0.968, Table 4). Calcium concentration was significantly higher in larvae fed the CP20 and CP16 diets compared to those larvae fed diets with lower protein contents (*p* = 0.003; R^2^ = 0.575, Table 4). Phosphorus concentration, however, showed no consistent trends across treatments. The calcium-to-phosphorus (Ca/P) ratio was highest in CP20-fed larvae and lowest in CP14-fed larvae (*p* = 0.014; R^2^ = 0.475, Table 4). Larval chitin concentration exhibited significant variation across the dietary treatments, with the lowest concentration observed in larvae fed the CP10 diet, in comparison to larvae fed diets with higher protein levels (*p* < 0.001; R^2^ = 0.718, Table 4). No significant effect of dietary protein levels was observed on glucosamine concentration in BSFL dry biomass (*p* = 0.799; R^2^ = 0.059, Table 4).

The sum of larval AA was affected in a quadratic manner by dietary protein levels, with the highest value observed in the CP14-fed larvae and the lowest in the CP10 group (*p* = 0.001; R^2^ = 0.796, Table 5). EAA, including arginine, histidine, isoleucine, leucine, lysine, methionine, phenylalanine, threonine, tryptophan, and valine, showed significant variation across dietary treatments. The CP14 diet generally resulted in the highest concentrations of these AA. Non-essential AA (NEAA) such as aspartic acid, glutamic acid, glycine, proline, and serine also varied significantly, with CP14 consistently showing the highest concentrations for aspartic acid, glutamic acid, and glycine (*p* < 0.001; R^2^ = 0.751 to 0.625, Table 5). Cysteine concentration in larvae dry biomass did not show any significant difference across treatments.

### 3.3. Deposition of Nutrients, Minerals, and Amino Acids

Water deposition was quadratically affected by dietary protein concentration, with the highest values observed in larvae fed the CP16 diet, while the larvae fed the CP 10 diet had the lowest deposition (*p* = 0.022; R^2^ = 0.601, Table 6). In contrast, gross energy deposition showed no significant variation across the treatments. Protein deposition was affected by the dietary treatments, with the highest deposition observed in larvae fed the CP14 diet, followed by those on the CP16 diet, compared to larvae fed the CP10 diet (*p* = 0.001; R^2^ = 0.851, Table 6). Fat deposition was highest in CP10-fed larvae, compared to larvae fed the CP14 and CP16 diets (*p* = 0.002; R^2^ = 0.624, Table 6). Chitin deposition was lowest in the CP10-fed larvae compared to those fed higher protein diets (*p* = 0.002; R^2^ = 0.594, Table 6). Ash deposition increased linearly with higher dietary protein concentrations, with the lowest deposition of ash in larvae fed with CP10 diet (*p* < 0.001; R^2^ = 0.961, Table 6). Calcium deposition was 35% higher in larvae fed the CP16 and CP20 diets compared to those fed the CP10 diet (*p* = 0.003; R^2^ = 0.575, Table 6). While larval glucosamine and phosphorus deposition showed no significant differences between the dietary treatments.

The deposition of EAA in BSFL was significantly influenced by dietary protein content. The lowest AA deposition was observed in larvae fed the CP10 diet, while higher deposition occurred in larvae fed diets with higher protein levels (*p* = 0.001; R^2^ = 0.815, Table 7). Most EAA, such as arginine, histidine, leucine, and valine, were deposited in the highest amounts in larvae fed the CP14 and CP16 diets, with the CP10 group showing the lowest values (*p* < 0.05, Table 7). Total AA deposition followed a quadratic pattern, with CP14 generally yielding the highest values (*p* < 0.001; R^2^ = 0.606 to 0.835, Table 7).

### 3.4. Retention Efficiency of Gross Energy, Nitrogen, and Amino Acids

Gross energy retention was higher in larvae fed the CP14 and CP16 diets compared to those on the CP10 diet (*p* = 0.001; R^2^ = 0.705, Table 8). Nitrogen retention also varied, with the highest levels observed in larvae fed the CP14 and CP10 diets, and the lowest in CP20-fed larvae (*p* < 0.001; R^2^ = 0.845, Table 8). Similarly, total AA retention was highest in larvae fed the CP14 and CP10 diets and lowest in larvae fed the CP20 diet (*p* < 0.001; R^2^ = 0.881, Table 8). Retention of EAA, including arginine, histidine, isoleucine, leucine, lysine, methionine, phenylalanine, threonine, tryptophan, and valine, was higher in larvae fed the CP14 diet compared to larvae fed the CP20 diet (*p* < 0.001; R^2^ = 0.625 to 0.902, Table 8). For NEAA, aspartic acid, glutamic acid, and glycine showed higher retention in larvae fed the CP14 diet, while cysteine and proline retention were also significantly higher in the CP10 and CP14 diets compared to the CP20 group (*p* < 0.05, Table 8).

## 4. Discussion

To our knowledge, no prior research has explored the effect of isoenergetic diets with varying protein concentrations on nutrient, mineral, and AA deposition and retention efficiency in *Hermetia illucens* larvae. This study provides novel insights in these nutritional aspects, identifying optimal dietary protein concentrations under isoenergetic conditions for rearing BSFL.

### 4.1. Growth Performance

In this study, dietary protein concentrations of 14% and 16% (CP14, CP16), with a gross energy content of 18.5 ± 0.3 MJ/kg dry matter, were identified as optimal for maximizing larval growth and biomass gain. These findings underscore the critical role of dietary protein in BSFL growth performance, aligning with previous research [11,12,13,31]. Oddon et al. [13] reported that dietary protein levels between 14% and 16% optimize larval development, aligning with our findings. In our study, a quadratic relationship between dietary protein content and larval performance was observed, suggesting that while adequate dietary protein is essential, dietary protein concentrations of 10% lead to decreased growth performance and biomass gain, as the AA supply becomes insufficient to support crucial metabolic and developmental functions. Nguyen et al. [20] found that dietary protein levels above a certain threshold do not increase larval biomass gain proportionally but reduce feed intake of larvae. This reduction in feed intake is likely a physiological response to the increased metabolic costs associated with processing excess dietary protein and a longer satiety. In larvae fed the 20% CP diet, a potential decreased feed intake may explain the lower body weights observed, as reduced intake limits the available nutrients for growth while the prolonged satiety further reduces overall nutrient consumption. Furthermore, BSFL reared on high-protein diets (32–50% crude protein, dry matter basis) showed elevated mortality rates [29,32]. Excess protein catabolism and excretion, resulting from diets with high protein levels, can lead to toxic metabolites, organ damage, and increased mitochondrial reactive oxygen species [29,31,32,33,34]. In Dipteran species, excess uric acid is converted to ammonium, contributing to harmful ammonia emissions [35]. The insulin-like growth factor (IGF) and TOR signaling pathways control body size, growth, and lifespan in insects, with nutrition being a key factor in these processes. In addition to IGF and TOR, the endocrine system, particularly through the hormones ecdysteroids and juvenile hormone, plays a vital role in regulating insect growth [20,21,36,37,38,39]. Proper dietary protein contents are essential for efficient larval growth and development, as excessive protein can interfere with these metabolic processes [39]. This suggests that a diet with 14% protein content and 18.5 MJ/kg dry matter may provide an optimal balance, fulfilling the larvae’s nutritional requirements while avoiding inefficiencies from excessive protein intake. The results of this study support the importance of balancing protein intake to promote larval growth performance and biomass production while reducing nitrogen loss as indicated by previous studies [12,13,14,15,40,41,42,43,44,45].

### 4.2. Bioconversion Efficiency

Larvae fed CP16 and CP14 diets exhibited the lowest feed conversion ratios (FCR) for both fresh and dry matter, indicating optimal feed-to-biomass conversion compared to other protein levels. This highlights the importance of balanced dietary protein in insect nutrition for efficient nutrient conversion and biomass gain [31,46,47]. Such efficiency enhances the economic viability of BSFL farming by reducing feed costs and increasing yields. However, protein requirements may vary with protein source quality, as shown in BSFL and other insects [18,48,49,50]. The gross energy conversion ratio (GECR) and nitrogen conversion ratio (NCR) also highlight the metabolic efficiency of larvae fed with optimal protein concentrations in their diet (14% and 16%). Seyedalmoosavi et al. [30] found a GECR ranging from 3.1 to 3.2 MJ/kg/100 g BSFL gain on a fresh matter basis, which is in line with the values observed in this study, ranging from 2.6 to 3.6. The observed differences in GECR values could also be influenced by variations in feed composition and the specific nutrient ratios in the diets used across studies. The lower NCR values observed in this study compared to those reported by Seyedalmoosavi et al. [30] could be attributed to differences in protein quality. Variations in the AA composition and bioavailability of the AA sources used in the diets may have led to less efficient nitrogen utilization in the study of Seyedalmoosavi et al. [30], whereas the protein sources used in this study may have supported more efficient nitrogen retention and metabolism. High NCR values in CP20-fed larvae suggest excessive protein intake leads to higher nitrogen excretion, consistent with findings by Pang et al. [47] and Gebremikael et al. [49], which note environmental concerns related to nitrogen pollution in insect farming. Excess nitrogen in the form of ammonia can lead to eutrophication and increased greenhouse gas emissions [47,48]. Variations in conversion parameters indicate that BSFL adapt their metabolism to dietary protein intake [50,51,52,53]. Insufficient protein intake (CP10) leads to impaired growth and potential nutritional stress. Conversely, excessive protein (CP20) results in diminishing returns, likely due to metabolic pathway saturation, wherein the capacity of the larvae metabolic systems to efficiently process and utilize the excess protein is surpassed, leading to inefficiencies in nutrient assimilation and increased excretion of nitrogen [23,45].

### 4.3. Body Composition, Deposition and Retention

The diet significantly influenced the body composition of BSFL, particularly affecting the larval protein and fat content. In terms of nutrient deposition and retention efficiency, the study found that BSFL utilize and store available nutrients differently based on the diet composition. CP14 was identified as the most optimal protein level because it resulted in the highest protein deposition while maintaining efficient feed conversion and nutrient retention. Although larvae fed CP16 exhibited similar performance in some parameters, increasing dietary protein beyond CP14 did not lead to proportional improvements in growth or nutrient utilization. Conversely, a diet with lower protein content (CP10), along with higher digestible carbohydrate contents, led to a decrease in protein synthesis and an increase in fat deposition [22,54,55].

When AA are sufficiently available, the larvae prioritize protein synthesis over fat storage, resulting in a reduction of fat content [16,22]. This is particularly significant in the production of high-quality BSFL protein meals for livestock nutrition, where the goal is to maximize protein content while minimizing the presence of ash and fat [5,6,7,8,9,10]. Additionally, this study found that the CP20 diet did not further increase protein levels in the larvae, suggesting diminishing returns from higher dietary protein concentrations, likely due to a lack of additional dietary energy sources.

The energy content of a diet is a critical determinant of larval metabolic efficiency and nutrient partitioning. Optimal dietary energy availability facilitates the efficient utilization of dietary protein, ensuring that amino acids are directed toward anabolic processes rather than catabolized for energy [36,56]. When the protein-to-energy ratio in diet is balanced, larvae prioritize protein synthesis for growth, enzymatic activity, and structural protein deposition, including cuticle components such as chitin, which are modulated by ecdysteroids [36]. Excess dietary energy relative to dietary protein intake shifts metabolism towards lipogenesis, resulting in increased larval fat deposition rather than enhanced larval protein retention [56]. Conversely, a dietary energy deficit can impair protein utilization, as amino acids are increasingly oxidized to meet energetic demands rather than being incorporated into larval biomass. In this study, diets were formulated with an energy concentration of 18.5 ± 0.3 MJ/kg dry matter, a level previously identified as optimal in BSFL nutrition. This dietary energy level ensures that dietary protein is efficiently utilized for growth rather than being diverted for maintenance metabolism, thereby supporting optimal larval development and biomass composition.

Chitin concentration was lowest in larvae fed the CP10 diet, suggesting that insufficient dietary protein limits chitin synthesis, a key structural component of the exoskeleton [50]. As chitin is composed of nitrogen-containing polysaccharides and derives partially from amino acid metabolism, a low-protein diet may restrict its biosynthesis due to limited dietary nitrogen and amino acids availability. Despite increased chitin concentration of larvae fed with higher-protein diets, glucosamine levels, a chitin biosynthesis derivative, showed no significant differences, indicating a consistent demand for structural integrity, influenced by BSFL development stage. This correlation between body weight and chitin deposition is not unique to BSFL, as analogous relationships exist in other livestock species, where skeletal development and structural protein accumulation, such as collagen and keratin formation, contribute to overall growth and body composition. However, the skeletal mass in pigs and poultry increases at a lower rate than muscle mass, resulting in a decreasing relative skeletal proportion as body weight increases. This is also consistent with the observations in this study, which found that chitin deposition increases with body mass, which may reflect its role in structural integrity and growth adaptation. Similarly, in crustaceans and fish with exoskeletal components, chitin accumulation increases with body size, emphasizing its role in structural reinforcement and growth adaptation [57]. Furthermore, the linear increase in ash and calcium concentrations in CP16- and CP20-fed larvae likely reflects a carry-over effect from the higher mineral content in protein-rich diets [19,30]. Additionally, residual dietary minerals in the digestive tract and increased mineral requirements for exoskeletal development may contribute to the elevated ash content. Thus, balancing dietary protein and dietary mineral content is essential for optimizing larval growth, nutrient retention efficiency, and biomass quality of BSFL.

### 4.4. Essential Amino Acid (EAA) Dynamics

When considering protein requirements, the quality and biological value of protein sources are crucial. Despite their importance, EAA are often overlooked in insect nutrition research, although they play a key role in optimizing waste digestion in BSFL production due to their varying nutrient profiles. Since animal cells cannot synthesize the carbon skeletons of EAA, these AA are crucial, as mentioned before, for synthesizing proteins required for survival, growth, development, and reproduction, including enzymes, structural proteins, transport proteins, immune proteins, and hormones [21,23,36,37,38,39]. The current study supports this by showing that feeds with limited AA content, such as CP10, result in reduced protein and AA deposition in larvae. EAA are essential for protein synthesis and other physiological functions, as noted by Tomberlin et al. [21], who highlighted the role of EAA in promoting efficient protein synthesis in BSFL. This relationship reflects the fine balance required to provide sufficient EAA for processes such as protein synthesis, exoskeleton, and enzymatic activity, without exceeding levels that could lead to inefficiencies or increased excretion. For example, methionine is essential for the synthesis of cysteine, which plays a critical role in the formation of structural proteins such as collagen and keratin in skin, hair, and feathers [58], and may also be involved in chitin synthesis. Similarly, the increased levels of leucine and lysine in the CP14 group compared to the CP10 diet indicate that this diet contains the building blocks for protein synthesis, which are required for larval growth and metamorphosis. While the larvae fed a CP14 diet had the highest total AA content in biomass, it is important to note that too much of one AA relative to others can cause an imbalance that affects growth and metabolism [23]. For example, an excess of lysine in the absence of sufficient methionine and threonine can reduce growth efficiency as the excess lysine cannot be fully utilized for protein synthesis [14,15,16]. Furthermore, the retention of EAA was strongly influenced by dietary protein concentration, with higher protein diets leading to enhanced retention of these critical micronutrients. This may suggest that a balanced intake of EAA is not only necessary for their deposition in larval biomass but also for maintaining optimal metabolic functions. Future research should further investigate these diet optimizations to standardize insect farming.

## 5. Conclusions

In this study, we assessed the effects of four different dietary crude protein levels on the growth performance, nutrient utilization, and retention of gross energy, nitrogen, and AA in BSFL. The highest larval performance was observed with 14% and 16% crude protein (CP), while the 10% CP diet resulted in the lowest productivity. A 14% CP diet optimized nutrient utilization, leading to the highest levels of larval protein, nitrogen, and AA, except for cysteine. Our findings indicate that insufficient dietary protein levels limit larval protein biosynthesis and negatively impacts growth performance, body composition, and the retention efficiency of nitrogen and AA.

## Figures and Tables

**Table 1:** Ingredients (g/kg), chemical composition (g/kg), and gross energy (MJ/kg) of the experimental diets.

Items	CON	CP10	CP14	CP16	CP20
Ingredients, g/kg					
Apple pomace	-	220.0	200.0	180.0	150.0
Rape seed cake	-	5.0	140.0	140.0	239.8
Wheat pulp	-	10.0	40.0	50.0	80.0
Biscuit flour	-	220.0	275.0	225.0	155.0
Bread crumbs	-	360.0	310.0	220.0	160.0
Rape seed oil	-	10.0	10.0	10.0	8.0
Wheat bran	500.0	175.0	175.0	175.0	175.0
Alfalfa	300.0	-	-	-	-
Maize	200.0	-	-	-	-
Analysed nutrient composition ^a^					
Dry matter	247	245	251	240	234
GE [MJ/kg]	16.4	18.5	18.5	18.3	18.8
Crude protein ^b^	142	102	144	163	202
N:GE ratio ^c^	1.36	0.89	1.26	1.43	1.72
Crude ash	63.0	52.6	55.6	56.9	60.3
Ether extract	24.3	72.3	70.6	68.2	62.2
aNDFom	370	237	306	313	361
Starch	187	312	260	254	220
Calcium	4.0	2.8	3.2	3.4	3.6
Phosphorus	6.8	3.5	5.5	5.7	6.9

Abbreviations: CON, control diet; CP10, 10% CP diet; CP14, 14% CP diet; CP16, 16% CP diet; CP20, 20% CP; GE, gross energy; aNDFom, amylase neutral detergent fiber organic matter. ^a^ Values are means of duplicate analyses. ^b^ Calculated as nitrogen × 6.25. ^c^ Calculated as nitrogen: gross energy (N:GE).

**Table 2:** Analyzed concentrations of amino acids of the experimental diets.

Items	CON	CP10	CP14	CP16	CP20
Total amino acids, g/kg DM	120.2	81.8	124.3	135.1	173.8
Essential amino acid composition, g/kg DM					
Arginine	7.8	4.4	7.1	7.6	10.1
Histidine	3.1	2.0	3.3	3.6	4.8
Isoleucine	5.4	3.5	5.4	5.9	7.6
Leucine	10.1	6.1	9.3	10.0	13.1
Lysine	6.9	3.6	6.0	6.1	8.8
Methionine	2.4	1.5	2.3	2.6	3.4
Phenylalanine	6.0	3.8	5.6	6.0	7.6
Threonine	5.4	3.5	5.5	6.1	8.2
Tryptophan	2.0	1.3	1.9	2.0	2.6
Valine	7.2	4.7	7.4	8.0	10.2
Non-essential amino acid composition, g/kg DM					
Alanine	8.3	5.2	8.0	8.4	10.7
Aspartic acid	11.1	7.6	10.1	11.1	13.6
Cysteine	2.2	1.7	2.9	3.2	4.2
Glutamic acid	19.8	16.2	24.5	27.3	34.3
Glycine	7.7	5.5	7.9	8.6	10.4
Proline	7.4	5.6	8.6	9.5	12.3
Serine	5.9	4.0	6.1	6.6	8.6

Abbreviations: CON, control diet; CP10, 10% CP diet; CP14, 14% CP diet; CP16, 16% CP diet; CP20, 20% CP.

**Table 3:** Growth performance and conversion parameters of *Hermetia illucens* larvae.

Parameter		Dietary Treatments				SE	*p*-Value
	CON	CP10	CP14	CP16	CP20		
Survival rate, %	97.5	90.4	94.6	93.8	92.7	0.04	0.677
Biomass gain ^1^, kg FM	1.63	1.51 ^c^	2.07 ^a^	2.15 ^a^	1.93 ^b^	5.40	<0.001
Biomass gain ^1^, kg DM	0.53	0.50 ^c^	0.65 ^a^	0.64 ^a^	0.58 ^b^	1.41	<0.001
FCR ^2^, FM	2.0	1.7 ^a^	1.2 ^c^	1.2 ^c^	1.4 ^b^	0.04	<0.001
FCR ^2^, DM	5.9	5.4 ^a^	4.2 ^c^	4.3 ^c^	4.7 ^b^	0.11	<0.001
GECR ^3^, FM	2.9	3.6 ^a^	2.6 ^b^	2.5 ^b^	2.9 ^b^	0.09	<0.001
GECR ^3^, DM	9.1	11.0 ^a^	8.4 ^b^	8.5 ^b^	9.3 ^b^	0.24	<0.001
NCR ^4^, FM	4.8	3.7 ^c^	3.9 ^b^	4.2 ^b^	5.7 ^a^	0.15	<0.001
NCR ^4^, DM	14.8	11.3 ^d^	12.6 ^cd^	14.1 ^bc^	18.9 ^a^	0.56	<0.001
TAACR ^5^, FM	21.4	15.8 ^c^	17.4 ^b^	18.3 ^b^	26.1 ^a^	0.77	<0.001
TAACR ^5^, DM	66.1	48.0 ^c^	55.5 ^b^	61.7 ^b^	86.8 ^a^	2.79	<0.001

^a–c^: Values in a row that are marked without the same superscript letter differ significantly (*p* < 0.05). Data are presented as means (*n* = 5/diet), standard error (SE), and *p*-value. Abbreviations: CON, control diet; CP10, 10% CP diet; CP14, 14% CP diet; CP16, 16% CP diet; CP20, 20% CP; FM, fresh matter; DM, dry matter. The control diet was not included in the statistical analysis. ^1^ Calculated as harvest weight per container (kg)—initial weight per container (kg). ^2^ FCR = Feed supply (g)/larval gain (g). ^3^ GECR = gross energy supply (MJ GE)/100 g larval gain. ^4^ NCR = nitrogen supply (g)/100 g larval gain. ^5^ TAACR = total amino acid supply (g)/100 g larval gain.

**Table 4:** Concentration of water, gross energy, nutrients, and minerals in biomass of *Hermetia illucens* larvae.

Item		Dietary Treatments				SE	*p*-Value
	CON	CP10	CP14	CP16	CP20		
Water, g/kg FM	684	680	693	708	704	2.89	0.082
GE, MJ/kg DM	23.6	27.0	26.7	27.2	27.7	1.13	0.183
Nutrients, g/kg DM							
Protein ^1^	377	305	365 ^a^	354 ^a^	348 ^ab^	7.05	<0.001
Fat	250	311 ^a^	271 ^c^	277 ^bc^	289 ^b^	4.59	<0.001
Chitin	63.6	58.2 ^b^	69.4 ^a^	67.7 ^a^	70.8 ^a^	2.89	<0.001
Glucosamine	1.5	1.5	1.6	1.3	1.7	0.11	0.799
Ash	102	67 ^c^	71 ^b^	74 ^b^	91 ^a^	2.84	<0.001
Minerals, g/kg DM							
Calcium	20.9	11.7 ^b^	11.9 ^b^	13.9 ^a^	15.4 ^a^	7.60	0.003
Phosphorus	9.2	6.2	6.6	6.3	6.8	2.52	0.135
Ca/P ratio, g/g DM	2.3	1.9 ^ab^	1.8 ^b^	2.2 ^ab^	2.3 ^a^	0.76	0.014

^a–c^: Values in a row that are marked without the same superscript letter differ significantly (*p* < 0.05). Data are presented as means (*n* = 5/diet), standard error (SE), and *p*-value. Abbreviations: CON, control diet; CP10, 10% CP diet; CP14, 14% CP diet; CP16, 16% CP diet; CP20, 20% CP; GE, gross energy. The control diet was not included in the statistical analysis. ^1^ Calculated as nitrogen × 4.76 (according to [17]).

**Table 5:** Concentration of amino acids in biomass of *Hermetia illucens* larvae.

Item		Dietary Treatments				SE	*p*-Value
	CON	CP10	CP14	CP16	CP20		
Total amino acids, g/kg DM							
Sum of AA	304.06	248.94 ^b^	307.46 ^a^	291.37 ^a^	282.03 ^a^	4.30	0.001
Essential amino acids, g/kg DM							
Sum of EAA	152.66	124.94 ^b^	157.03 ^a^	148.04 ^a^	142.25 ^a^	2.04	<0.001
Arginine	14.93	12.86 ^b^	16.68 ^a^	15.26 ^a^	14.58 ^a^	0.48	0.033
Histidine	9.41	7.51 ^b^	10.46 ^a^	10.16 ^a^	9.35 ^a^	0.27	0.001
Isoleucine	15.20	13.00 ^b^	15.65 ^a^	14.58 ^a^	14.19 ^ab^	0.22	<0.001
Leucine	27.45	23.65 ^c^	28.29 ^a^	26.67 ^ab^	25.81 ^bc^	0.40	0.001
Lysine	21.99	16.48 ^b^	21.10 ^a^	20.02 ^a^	19.43 ^a^	0.44	<0.001
Methionine	6.13	5.02 ^c^	6.36 ^a^	5.92 ^ab^	5.73 ^b^	0.10	<0.001
Phenylalanine	14.47	11.24 ^b^	14.25 ^a^	13.39 ^a^	12.85 ^ab^	0.28	0.001
Threonine	13.51	11.13 ^c^	13.78 ^a^	13.03 ^ab^	12.53 ^b^	0.21	<0.001
Tryptophan	7.48	5.88 ^b^	7.76 ^a^	7.70 ^a^	7.14 ^a^	0.19	<0.001
Valine	22.09	18.17 ^c^	22.70 ^a^	21.31 ^ab^	20.64 ^b^	0.37	<0.001
Non-essential amino acids, g/kg DM							
Sum of NEAA	151.40	124.00 ^b^	150.43 ^a^	143.33 ^a^	139.78 ^a^	1.82	<0.001
Alanine	25.5	19.87	23.86	22.91	22.28	0.62	0.222
Aspartic acid	30.6	24.09 ^b^	31.93 ^a^	30.04 ^a^	28.93 ^a^	0.65	<0.001
Cysteine	2.7	2.02	2.31	2.25	2.38	0.06	0.065
Glutamic acid	36.6	30.13 ^b^	35.72 ^a^	33.78 ^ab^	33.35 ^ab^	0.51	0.001
Glycine	20.6	16.56 ^b^	20.69 ^a^	19.81 ^a^	19.21 ^a^	0.39	0.001
Proline	21.6	19.24 ^b^	21.50 ^a^	20.85 ^a^	20.55 ^ab^	0.26	0.002
Serine	13.8	12.09 ^b^	14.42 ^a^	13.69 ^ab^	13.08 ^ab^	0.23	0.005

^a–c^: Values in a row that are marked without the same superscript letter differ significantly (*p* < 0.05); Data are presented as means (*n* = 5/diet), standard error (SE), and *p*-value. Abbreviations: CON, control diet; CP10, 10% CP diet; CP14, 14% CP diet; CP16, 16% CP diet; CP20, 20% CP. The control diet was not included in the statistical analysis.

**Table 6:** Deposition of water, energy, nutrients, and minerals per 1000 larvae of *Hermetia illucens* larvae.

Item		Dietary Treatments				SE	*p*-Value
	CON	CP10	CP14	CP16	CP20		
Water, g FM/d	10.3	10.1 ^b^	13.5 ^ab^	14.5 ^a^	13.1 ^ab^	0.39	0.022
GE, MJ kg DM/d	0.12	0.15	0.16	0.16	0.16	0.05	0.122
Nutrients, g DM/d							
Protein ^1^	1.85	1.50 ^b^	2.25 ^a^	2.16 ^a^	1.97 ^ab^	0.08	0.001
Fat	1.26	1.78 ^a^	1.60 ^b^	1.62 ^b^	1.69 ^ab^	0.04	0.002
Chitin	0.30	0.27 ^b^	0.35 ^a^	0.35 ^a^	0.42 ^a^	0.02	0.002
Ash	0.50	0.32 ^b^	0.44 ^a^	0.45 ^a^	0.51 ^a^	0.01	<0.001
Glucosamine mg DM/d	7.3	7.6	8.5	8.2	8.7	0.65	0.799
Minerals, mg DM/d							
Calcium	100.9	57.9 ^c^	72.9 ^b^	84.3 ^a^	86.9 ^a^	3.42	0.004
Phosphorus	44.9	30.6	40.7	38.2	38.3	1.32	0.135

^a–c:^ Values in a row that are marked without the same superscript letter differ significantly (*p* < 0.05). Data are presented as means (*n* = 5/diet), standard error (SE), and *p*-value. Abbreviations: CON, control diet; CP10, 10% CP diet; CP14, 14% CP diet; CP16, 16% CP diet; CP20, 20% CP. The control diet was not included in the statistical analysis. ^1^ Calculated as nitrogen × 4.76 according to [17]. Deposition = Biomass gain (g/d) × nutrient (g/kg)/1000 larvae.

**Table 7:** Deposition of amino acids per 1000 larvae of *Hermetia illucens* larvae.

Item		Dietary Treatments				SE	*p*-Value
	CON	CP10	CP14	CP16	CP20		
Total amino acids, g DM/d							
Sum of AA	1.45	1.19 ^b^	1.84 ^a^	1.74 ^a^	1.65 ^a^	0.05	0.001
Essential amino acids, mg DM/d							
Arginine	73.1	63.0 ^b^	102.5 ^a^	92.7 ^a^	87.1 ^a^	4.02	0.002
Histidine	46.1	36.8 ^b^	64.3 ^a^	61.5 ^a^	52.7 ^ab^	2.45	<0.001
Isoleucine	74.1	63.8 ^b^	96.0 ^a^	88.2 ^a^	79.9 ^ab^	2.63	<0.001
Leucine	134.0	116.0 ^b^	173.6 ^a^	161.4 ^a^	145.4 ^ab^	4.90	<0.001
Lysine	107.1	80.8 ^b^	129.4 ^a^	121.1 ^a^	109.5 ^ab^	3.88	<0.001
Methionine	29.9	24.6 ^b^	39.0 ^a^	35.8 ^a^	32.3 ^a^	1.14	<0.001
Phenylalanine	70.4	55.1 ^b^	87.3 ^a^	81.0 ^a^	72.4 ^ab^	2.55	<0.001
Threonine	65.9	54.6 ^b^	84.5 ^a^	78.9 ^a^	70.6 ^ab^	2.41	<0.001
Tryptophan	36.3	28.9 ^b^	47.6 ^a^	46.7 ^a^	40.1 ^a^	1.70	<0.001
Valine	107.7	89.2 ^b^	139.2 ^a^	129.0 ^a^	116.2 ^a^	4.01	<0.001
Non-essential amino acids, mg DM/d							
Alanine	123.8	97.7 ^b^	146.0 ^a^	138.2 ^a^	125.3 ^a^	4.43	0.004
Aspartic acid	149.4	118.0 ^b^	196.0 ^a^	181.8 ^a^	163.0 ^ab^	6.38	<0.001
Cysteine	13.2	9.9 ^b^	14.1 ^a^	13.6 ^a^	13.4 ^a^	0.38	<0.001
Glutamic acid	178.7	147.8 ^b^	210.0 ^a^	204.3 ^a^	187.7 ^a^	5.72	<0.001
Glycine	100.6	81.2 ^b^	127.0 ^a^	120.0 ^a^	108.2 ^ab^	3.93	<0.001
Proline	105.6	94.3 ^b^	131.9 ^a^	126.1 ^a^	115.5 ^a^	3.46	<0.001
Serine	67.3	59.3 ^b^	88.5 ^a^	83.0 ^a^	73.7 ^a^	2.64	<0.001

^a–b^: Values in a row that are marked without the same superscript letter differ significantly (*p* < 0.05). Data are presented as means (*n* = 5/diet), standard error (SE), and *p*-value. Abbreviations: CON, control diet; CP10, 10% CP diet; CP14, 14% CP diet; CP16, 16% CP diet; CP20, 20% CP. The control diet was not included in the statistical analysis.

**Table 8:** Retention (in %) of gross energy, nitrogen, and amino acids of *Hermetia illucens* larvae.

Item		Dietary Treatments				SE	*p*-Value
	CON	CP10	CP14	CP16	CP20		
Gross energy	27.1	25.0 ^b^	32.2 ^a^	32.6 ^a^	29.7 ^a^	0.74	0.001
Nitrogen	53.8	56.9 ^ab^	61.6 ^a^	53.3 ^b^	39.1 ^c^	1.69	<0.001
Total amino acids	48.3	54.5 ^ab^	58.0 ^a^	49.3 ^b^	33.9 ^c^	1.82	<0.001
Essential amino acids, mg/d							
Arginine	36.0	51.3 ^a^	54.6 ^a^	45.6 ^a^	30.1 ^b^	2.33	<0.001
Histidine	55.6	66.2 ^a^	72.9 ^a^	64.1 ^a^	40.6 ^b^	2.58	<0.001
Isoleucine	52.9	65.8 ^a^	67.9 ^a^	56.3 ^b^	39.2 ^c^	2.27	<0.001
Leucine	51.5	68.6 ^ab^	71.0 ^a^	60.3 ^b^	41.1 ^c^	2.41	<0.001
Lysine	67.8	80.1 ^a^	82.1 ^a^	75.2 ^a^	46.1 ^b^	2.88	<0.001
Methionine	49.0	60.5 ^ab^	63.3 ^a^	52.7 ^b^	35.2 ^c^	2.16	<0.001
Phenylalanine	45.2	52.1 ^a^	58.8 ^a^	50.4 ^a^	35.6 ^b^	1.77	<0.001
Threonine	47.5	56.6 ^a^	57.8 ^a^	48.2 ^b^	32.0 ^c^	2.00	<0.001
Tryptophan	71.1	80.1 ^a^	92.8 ^a^	87.8 ^a^	57.6 ^b^	3.10	<0.001
Valine	58.0	68.4 ^ab^	71.6 ^a^	60.5 ^b^	42.1 ^c^	2.29	<0.001
Non-essential amino acids, mg/d							
Alanine	57.6	68.1 ^a^	69.5 ^a^	61.8 ^a^	43.5 ^b^	2.53	0.003
Aspartic acid	52.2	56.0 ^b^	73.3 ^a^	61.5 ^b^	44.3 ^c^	2.20	<0.001
Cysteine	23.7	21.2 ^a^	18.6 ^ab^	16.1 ^b^	11.7 ^c^	0.97	<0.001
Glutamic acid	34.9	33.0 ^a^	33.9 ^a^	28.2 ^b^	20.3 ^c^	2.20	<0.001
Glycine	50.3	53.6 ^a^	60.8 ^a^	52.4 ^a^	38.5 ^b^	1.70	<0.001
Proline	55.2	60.6 ^a^	57.9 ^a^	49.7 ^b^	34.9 ^c^	2.04	<0.001
Serine	44.2	43.0 ^b^	57.8 ^a^	47.0 ^b^	31.6 ^c^	1.84	<0.001

^a–c^: Values in a row that are marked without the same superscript letter differ significantly (*p* < 0.05). Data are presented as means (*n* = 5/diet), standard error (SE), and *p*-value. Abbreviations: CON, control diet; CP10, 10% CP diet; CP14, 14% CP diet; CP16, 16% CP diet; CP20, 20% CP. The control diet was not included in the statistical analysis.

## Data Availability

The datasets used and analyzed during the current study are available from the corresponding author on reasonable request.

## Abbreviations

AA: amino acids; BSFL: black soldier fly larvae; CP: crude protein; DM: dry matter; DOL: day-old-larvae; EAA: essential amino acids; FM: fresh matter; FCR: feed conversion ratio; GE: gross energy; GECR: gross energy conversion ratio; N: nitrogen; NCR: nitrogen conversion ratio; aNDFom: amylase neutral detergent fiber organic matter; NEAA: non-essential amino acids; SE: standard error; TAACR: total amino acid conversion ratio.

## References

[1] FAO (2017). The Future of Food and Agriculture—Trends and Challenges.

[2] Wu G., Fanzo J., Miller D.D., Pingali P., Post M., Steiner J.L., Thalacker-Mercer A.E. (2014). Production and supply of high-quality food protein for human consumption: Sustainability, challenges, and innovations. Ann. N. Y. Acad. Sci..

[3] Diener S., Solano N.M.S., Gutiérrez F.R., Zurbrügg C., Tockner K. (2011). Biological treatment of municipal organic waste using black soldier fly larvae. Waste Biomass Valoriz..

[4] Surendra K.C., Olivier R., Tomberlin J.K., Jha R., Khanal S.K. (2016). Bioconversion of organic wastes into biodiesel and animal feed via insect farming. Renew. Energy.

[5] Bosch G., Swanson K. (2021). Effect of using insects as feed on animals: Pet dogs and cats. J. Insects Food Feed.

[6] Dörper A., Veldkamp T., Dicke M. (2021). Use of black soldier fly and house fly in feed to promote sustainable poultry production. J. Insects Food Feed.

[7] Hartinger K., Fröschl K., Ebbing M.A., Bruschek-Pfleger B., Schedle K., Schwarz C., Gierus M. (2022). Suitability of *Hermetia illucens* larvae meal and fat in broiler diets: Effects on animal performance, apparent ileal digestibility, gut histology, and microbial metabolites. J. Anim. Sci. Biotechnol..

[8] Hartinger K., Greinix J., Thaler N., Ebbing M.A., Yacoubi N., Schedle K., Gierus M. (2021). Effect of graded substitution of soybean meal by *Hermetia illucens* larvae meal on animal performance, apparent ileal digestibility, gut histology, and microbial metabolites of broilers. Animals.

[9] Heuel M., Sandrock C., Leiber F., Mathys A., Gold M., Zurbrügg C., Gangnat I., Kreuzer M., Terranova M. (2021). Black soldier fly larvae meal and fat can completely replace soybean cake and oil in diets for laying hens. Poult. Sci..

[10] Alvanou M.V., Kyriakoudi A., Makri V., Lattos A., Feidantsis K., Papadopoulos D.K., Georgoulis I., Apostolidis A.P., Michaelidis B., Mourtzinos I. (2023). Effects of dietary substitution of fishmeal by black soldier fly (*Hermetia illucens*) meal on growth performance, whole-body chemical composition, and fatty acid profile of *Pontastacus leptodactylus* juveniles. Front. Physiol..

[11] Barragan-Fonseca K., Dicke M., Van Loon J. (2017). Nutritional value of the black soldier fly (*Hermetia illucens* L.) and its suitability as animal feed—A review. J. Insects Food Feed.

[12] Cammack J., Tomberlin J. (2017). The impact of diet protein and carbohydrate on select life-history traits of the black soldier fly *Hermetia illucens* (L.) (Diptera: Stratiomyidae). Insects.

[13] Oddon S.B., Biasato I., Gasco L. (2022). Isoenergetic-practical and semi-purified diets for protein requirement determination in *Hermetia illucens* larvae: Consequences on life history traits. J. Anim. Sci. Biotechnol..

[14] Davis G.R.F. (1975). Essential dietary amino acids for growth of larvae of the yellow mealworm, *Tenebrio molitor* L.. J. Nutr..

[15] Chang C.L. (2004). Effect of amino acids on larvae and adults of *Ceratitis capitata*, Diptera: Tephritidae. Ann. Entomol. Soc. Am..

[16] Lee K.P. (2007). The interactive effects of protein quality and macronutrient imbalance on nutrient balancing in an insect herbivore. J. Exp. Biol..

[17] Janssen R.H., Vincken J., Van Den Broek L.A.M., Fogliano V., Lakemond C.M.M. (2017). Nitrogen-to-protein conversion factors for three edible insects: *Tenebrio molitor*, *Alphitobius diaperinus*, and *Hermetia illucens*. J. Agric. Food Chem..

[18] Gold M., Cassar C.M., Zurbrügg C., Kreuzer M., Boulos S., Diener S., Mathys A. (2020). Biowaste treatment with black soldier fly larvae: Increasing performance through the formulation of biowastes based on protein and carbohydrates. Waste Manag..

[19] Spranghers T., Ottoboni M., Klootwijk C., Ovyn A., Deboosere S., De Meulenaer B., Michiels J., Eeckhout M., De Clercq P., De Smet S. (2016). Nutritional composition of black soldier fly (*Hermetia illucens*) prepupae reared on different organic waste substrates. J. Sci. Food Agric..

[20] Nguyen T.T.X., Tomberlin J.K., Vanlaerhoven S. (2013). Influence of resources on *Hermetia illucens* (Diptera: Stratiomyidae) larval development. J. Med. Entomol..

[21] Tomberlin J.K., Miranda C., Flint C., Harris E., Wu G. (2023). Nutrients limit production of insects for food and feed: An emphasis on nutritionally essential amino acids. Anim. Front..

[22] Wu G., Li P. (2022). The “ideal protein” concept is not ideal in animal nutrition. Exp. Biol. Med..

[23] Lemme A., Klüber P. (2024). Rethinking amino acid nutrition of black soldier fly larvae (*Hermetia illucens*) based on insights from an amino acid reduction trial. Insects.

[24] Oonincx D.G.A.B., Van Broekhoven S., Van Huis A., Van Loon J.J.A. (2015). Feed conversion, survival and development, and composition of four insect species on diets composed of food by-products. PLoS ONE.

[25] Hogsette J.A. (1992). New diets for production of house flies and stable flies (Diptera: Muscidae) in the laboratory. J. Econ. Entomol..

[26] Verband Deutscher Landwirtschaftlicher Untersuchungs- und Forschungsanstalten (VDLUFA) (2007). Die Chemische Untersuchung von Futtermitteln.

[27] Wang M., Toghyani M., Macelline S.P., Liu S.Y. (2025). Sorghum surpasses wheat as a feed grain for broiler chickens following dietary crude protein reductions. J. Anim. Sci. Biotechnol..

[28] Urs M.J., Moerschbacher B.M., Cord-Landwehr S. (2023). Quantitative enzymatic-mass spectrometric analysis of the chitinous polymers in fungal cell walls. Carbohydr. Polym..

[29] Guillaume J.B., Mezdour S., Marion-Poll F., Terrol C., Schmidely P. (2023). Asymptotic Estimated Digestibility, a New Indicator of Black Soldier Fly (*Hermetia illucens*) Conversion Efficiency in Relation to Larval Density. J. Insects Food Feed.

[30] Seyedalmoosavi M.M., Mielenz M., Schleifer K., Görs S., Wolf P., Tränckner J., Hüther L., Dänicke S., Daş G., Metges C.C. (2023). Upcycling of recycled minerals from sewage sludge through black soldier fly larvae: Impact on growth and mineral accumulation. J. Environ. Manag..

[31] Eggink K.M., Donoso I.G., Dalsgaar J. (2023). Optimal dietary protein to carbohydrate ratio for black soldier fly (*Hermetia illucens*) larvae. J. Insects Food Feed.

[32] Tschirner M., Simon A. (2015). Influence of different growing substrates and processing on the nutrient composition of black soldier fly larvae destined for animal feed. J. Insects Food Feed.

[33] Ayala V., Naudí A., Sanz A., Caro P., Portero-Otin M., Barja G., Pamplona R. (2007). Dietary protein restriction decreases oxidative protein damage, peroxidizability index, and mitochondrial complex I content in rat liver. J. Gerontol. A Biol. Sci. Med. Sci..

[34] Lee K.P., Simpson S.J., Clissold F.J., Brooks R., Ballard J.W.O., Taylor P.W., Soran N., Raubenheimer D. (2008). Lifespan and reproduction in Drosophila: New insights from nutritional geometry. Proc. Natl. Acad. Sci. USA.

[35] Green T.R., Popa R. (2012). Enhanced ammonia concentration in compost leachate processed by black soldier fly larvae. Appl. Biochem. Biotechnol..

[36] Hawkey K.J., Lopez-Viso C., Brameld J.M., Parr T., Salter A.M. (2021). Insects: A potential source of protein and other nutrients for feed and food. Annu. Rev. Anim. Biosci..

[37] Dabour N., Bando T., Nakamura T., Miyawaki K., Mito T., Ohuchi H., Noji S. (2011). Cricket body size is altered by systemic RNAi against insulin signaling components and epidermal growth factor receptor. Dev. Growth Differ..

[38] Oldham S., Hafen E. (2003). Insulin/IGF and target of rapamycin signaling: A TOR de force in growth control. Trends Cell Biol..

[39] Mirth C.K., Riddiford L.M. (2007). Size assessment and growth control: How adult size is determined in insects. BioEssays.

[40] Danieli N., Lussiana N., Gasco N., Amici N., Ronchi N. (2019). The effects of diet formulation on the yield, proximate composition, and fatty acid profile of the black soldier fly (*Hermetia illucens* L.) prepupae intended for animal feed. Animals.

[41] Nyakeri E., Ayieko M., Amimo F., Salum H., Ogola H. (2019). An optimal feeding strategy for black soldier fly larvae biomass production and faecal sludge reduction. J. Insects Food Feed.

[42] Ewald N., Vidakovic A., Langeland M., Kiessling A., Sampels S., Lalander C. (2020). Fatty acid composition of black soldier fly larvae (*Hermetia illucens*)—Possibilities and limitations for modification through diet. Waste Manag..

[43] Seyedalmoosavi M.M., Mielenz M., Veldkamp T., Daş G., Metges C.C. (2022). Growth efficiency, intestinal biology, and nutrient utilization and requirements of black soldier fly (*Hermetia illucens*) larvae compared to monogastric livestock species: A review. J. Anim. Sci. Biotechnol..

[44] Meneguz M., Schiavone A., Gai F., Dama A., Lussiana C., Renna M., Gasco L. (2018). Effect of rearing substrate on growth performance, waste reduction efficiency and chemical composition of black soldier fly (*Hermetia illucens*) larvae. J. Sci. Food Agric..

[45] Spranghers T., Moradei A., Vynckier K., Boudrez M., Pinotti L., Ottoboni M. (2024). Amino acid requirements of yellow mealworm and black soldier fly larvae. J. Insects Food Feed.

[46] Altaye S.Z., Pirk C.W., Crewe R.M., Nicolson S.W. (2010). Convergence of carbohydrate-biased intake targets in caged worker honeybees fed different protein sources. J. Exp. Biol..

[47] Pang W., Hou D., Chen J., Nowar E.E., Li Z., Hu R., Tomberlin J.K., Yu Z., Li Q., Wang S. (2020). Reducing greenhouse gas emissions and enhancing carbon and nitrogen conversion in food wastes by the black soldier fly. J. Environ. Manag..

[48] Gebremikael M.T., Van Wickeren N., Hosseini P.S., De Neve S. (2022). The impacts of Black Soldier Fly Frass on nitrogen availability, microbial activities, C sequestration, and plant growth. Front. Sustain. Food Syst..

[49] Purkayastha D., Sarkar S. (2021). Sustainable waste management using black soldier fly larva: A review. Int. J. Environ. Sci. Technol..

[50] Liu X., Chen X., Wang H., Yang Q., Rehman K.U., Li W., Cai M., Li Q., Mazza L., Zhang J. (2017). Dynamic changes of nutrient composition throughout the entire life cycle of black soldier fly. PLoS ONE.

[51] Gligorescu A., Chen L., Jensen K., Moghadam N.N., Kristensen T.N., Sørensen J.G. (2023). Rapid Evolutionary Adaptation to Diet Composition in the Black Soldier Fly (*Hermetia illucens*). Insects.

[52] Raman S.S., Stringer L.C., Bruce N.C., Chong C.S. (2022). Opportunities, challenges and solutions for black soldier fly larvae-based animal feed production. J. Clean. Prod..

[53] Ravi H.K., Degrou A., Costil J., Trespeuch C., Vian M.A., Chemat F., Vian M.A. (2020). Larvae Mediated Valorization of Industrial, Agriculture and Food Wastes: Biorefinery Concept through Bioconversion, Processes, Procedures, and Products. Processes.

[54] Barragan-Fonseca K., Gort G., Dicke M., Van Loon J. (2021). Nutritional plasticity of the black soldier fly (*Hermetia illucens*) in response to artificial diets varying in protein and carbohydrate concentrations. J. Insects Food Feed.

[55] Pimentel A.C., Montali A., Bruno D., Tettamanti G. (2017). Metabolic adjustment of the larval fat body in *Hermetia illucens* to dietary conditions. J. Asia-Pac. Entomol..

[56] Tognocchi M., Abenaim L., Adamaki-Sotiraki C., Athanassiou G.C., Rumbos I.C., Mele M., Conti B., Conte G. (2024). Effect of different diet composition on the fat profile of two different black soldier fly larvae populations. Animal.

[57] Olatunji O. (2024). Chitin. Aquatic Biopolymers.

[58] Giteru S.G., Ramsey D.H., Hou Y., Cong L., Mohan A., Bekhit A.E.A. (2022). Wool keratin as a novel alternative protein: A comprehensive review of extraction, purification, nutrition, safety, and food applications. Compr. Rev. Food. Sci. Food. Saf..

